# BloomSec: Scalable and privacy-preserving searchable encryption for cloud environments

**DOI:** 10.1371/journal.pone.0336944

**Published:** 2025-12-09

**Authors:** Abdul Nasir Khan, Ayesha Naveed, Abid Mehmood, Deepak Arora, Atta ur Rehman Khan, Javid Ali

**Affiliations:** 1 COMSATS University Islamabad, Abbottabad Campus, Abbottabad, Pakistan; 2 Abu Dhabi University, Abu Dhabi, United Arab Emirates; 3 Liwa College, Abu Dhabi, United Arab Emirates; 4 College of Engineering and IT, Ajman University, Ajman, United Arab Emirates; Oakland University, UNITED STATES OF AMERICA

## Abstract

The utilization of on-demand remote cloud services provides a flexible way to fulfill the demands of emerging resource-intensive applications. However, migrating data to the cloud also introduced security threats, including unauthorized access and information theft. To resolve this issue, the existing solutions encrypt information locally before uploading it to the server. This process provides information protection with the limitation of non-searchable data. To overcome this limitation, searchable encryption has emerged as a promising cryptographic technique. Some existing searchable encryption techniques are facing data leakage issues by exposing search queries or data to the cloud service provider. Another class of existing searchable schemes introduces processing cost or communication overhead for the data user. The recent searchable solution that is both secure and efficient for data users is Labeled Searchable Encryption (LSE). However, LSE cannot manage large datasets effectively and introduces communication overhead on the data user side. To ensure that Secure Searchable Encryption (SSE) can meet the demands of modern data-driven applications without compromising security and performance, this study aims to investigate and develop novel approaches to enhance the efficiency and security of SSE for large datasets. Experimental findings have proved that the proposed BloomSec is much more efficient and scalable than the classic method of Labeled Searchable Encryption (LSE), consuming significantly less overhead for users, which makes it practically useful for a large dataset without compromising security.

## 1 Introduction

**Fig 1:**
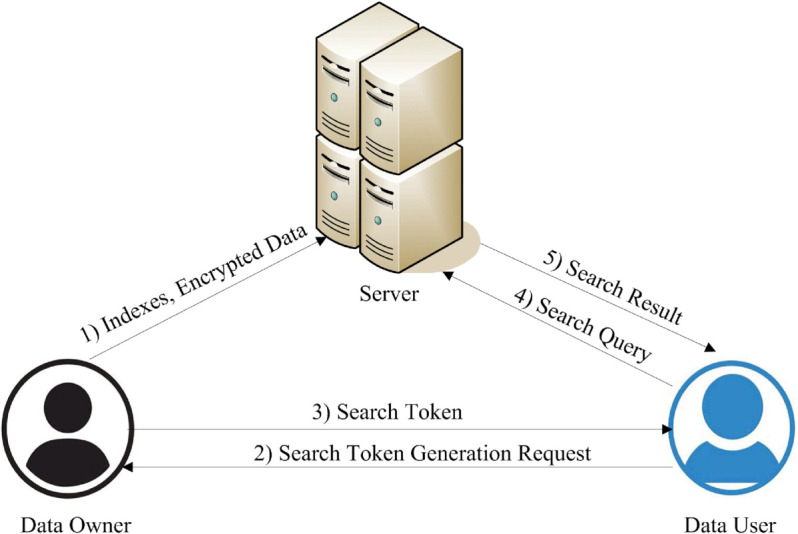
Searchable encryption system model.

A cryptographic technique called Searchable Encryption (SE) enables individuals to locate encrypted data without decrypting it beforehand. This approach is particularly important for remote services where confidentiality and safety of information are critical. SE preserves the confidentiality of private information while allowing people to search it [1]. The searchable encryption system needs three essential parts: (a) the Data User (DU), (b) the cloud server, and the (c) Data Owner (DO). Fig 1 shows the general system model of searchable encryption.

The data owner is responsible for constructing the protected search indexes from the keywords of the file. Afterward, the search indexes, corresponding file identifiers, and encrypted files are uploaded to the remote cloud server. Data users are individuals who intend to access and use data stored in the cloud by the data owner [2]. A cloud server is a machine that offers storage to parties involved and search functionality to both data users and owners. The data user initiates a search by submitting a query, which is then transformed into a search token before being sent to the cloud server to search for the encrypted data [3]. The cloud server compares the received search token with the stored search indexes. If a match is found, it returns the relevant encrypted document identifiers. As the cloud server only processes tokens, indexes, and encrypted data, this process does not reveal any information about the search query or the underlying data to the cloud.

The increasing dependence on cloud systems for data recovery and storage has made privacy protection crucial. To ensure security and prevent unauthorized access, important data must be encrypted before being stored on the remote servers. While encryption safeguards information, it complicates some tasks, such as searching on encrypted data [4]. Finding the ideal balance between protection and efficient search capabilities is necessary to guarantee the confidentiality of information and its availability in cloud systems. There are various approaches that make the encrypted content searchable. The most widely used approaches include (a) symmetric searchable encryption, (b) asymmetric searchable encryption, and (c) attribute-based searchable encryption.

Symmetric Searchable Encryption (SSE) utilizes the same key for encryption as well as for decryption [5]. SSE enables individuals to search for a specific keyword stored in encrypted form on a cloud server. Users can search over encrypted data without prior decrypting [3]. SSE provides robust safety protections by allowing only the individuals who have the secret key to create a search query and decrypt the result. Another advantage of searchable encryption is that its computational overhead is low. Along with the advantages, there are a few disadvantages, including the challenges in key management. SSE requires only one key for both encryption and decryption of the data; if the key is disclosed, then the data is compromised. It works fast but requires secure handling of keys. Similarly, Asymmetric Searchable Encryption (ASE) utilizes a combination of public key and private key to allow keyword searches against encrypted data. A private key is used to generate search tokens and decrypt the result [5]. The role of the public key is to encrypt the content and keywords. Even though ASE ensures strong confidentiality, its computational cost limits its use with huge datasets or frequent modifications. Because key management also becomes more complicated, users must monitor and maintain key pairs for secure access and searches. Likewise, keyword searches over encrypted content with fine-grained access controls are made possible by Attribute-Based Searchable Encryption (ABSE) [6], which combines attribute-based access control and searchable encryption. Some characteristics are related to both the data and the keyword all over the encryption process [7]. Access is only provided to users whose characteristics match the defined policy, and each user’s characteristics are linked to a private key. ABSE offers data privacy and selective access, but it also has complicated key management and a high computational cost. Large systems with many functions can benefit greatly from it, but applications that require frequent searches will find it less scalable [8,9].

When compared with SSE, ASE, and ABSE, SSE is unquestionably the most practical and effective option. SSE is well-suited for situations where performance is crucial, as it takes advantage of efficient symmetric cryptography, such as Advanced Encryption Standard (AES), to provide efficient cryptographic operations [10]. In contrast, ASE employs costly public-key processes that lead to substantial processing overhead, especially when handling large datasets [11].

This research study focuses on the SSE technique because of its advantages over ASE and ABSE, as highlighted earlier. The main contributions of this research study are:

- Critically analyzing the current state-of-the-art symmetric searchable encryption techniques
- To identify the challenges and limitations of the existing SSE.
- To propose a new symmetric searchable encryption technique that incorporates a bloom filter, overcoming the limitations of existing methods
- To compare the proposed SSE and the current state-of-the-art technique in terms of storage cost, communication cost, and operational time.

The rest of the paper is organized as follows: Sect 2 discusses the related work on symmetric searchable encryption schemes, Sect 3 contains the proposed symmetric searchable encryption scheme, Sect 4 compares the proposed scheme with the existing scheme based on evaluation parameters, and Sect 5 concludes the research.

## 2 Literature review

The existing SSE schemes can be categorized as (a) single keyword search, (b) multi-keyword search, (c) range search, and (d) boolean search. In a single keyword search, the data owner creates search indexes of keywords extracted from the dataset [12]. The search indexes are uploaded along with the encrypted file to the remote/cloud server. The schemes in this category enable the data owner/user to search for only a single keyword within encrypted files stored on the remote/cloud server. During the search process, the data owner/user generates a token of the search query and forwards it to a remote server to perform a search over all the encrypted files [13]. Single keyword search schemes are fast, easy to implement, and require simple indexing, yet lack flexibility and offer limited functionality. Multi-keyword search schemes allow data users/owners to search multiple keywords simultaneously without revealing the information and queries to the server [14]. Multi-dimensional indexing [15,16], bitmap indexing [17–19], and secure KNN [20,21] approaches are used for creating indexes of the dataset that can handle data with multiple attributes or dimensions. Although multi-keyword search schemes provide flexibility in search, they are computationally intensive and difficult to implement. Range search schemes allow the data owner/user to search data within a specific range, for example, finding data between 2021 and 2024 [22]. These schemes are ideal for searching numerical or time-based data in encrypted files, but they are computationally intensive, require sophisticated indexing, and do not perform well for text-heavy documents. Boolean search schemes allow the data user/owner to perform a Boolean operator (OR, AND, NOT) on a search query [23,24]. This helps the data owner/user to execute the complex search query that satisfy specific logical conditions [25]. Boolean search schemes deal with multiple logical operators, which require sophisticated infrastructure.

In this study, we focus on single-keyword search schemes. These single-keyword searchable symmetric encryption schemes can be classified into: (a) bloom filter-based SSE techniques, and (b) non–bloom filter-based SSE techniques.

The existing Bloom filter-based symmetric searchable encryption schemes use probabilistic bloom filters to generate the search index based on the keywords of the encrypted files. The authors in [26] proposed a searchable symmetric encryption scheme for smart grid data. The proposed scheme allows search on encrypted storage in untrusted cloud environments. Search indexes are constructed based on pseudo-random functions and bloom filters to facilitate efficient keyword search over encrypted data. However, the proposed approach also faces a high false positive rate and data leakage issues from the negligent selection of pseudo-random functions. The limitations of the proposed approach are addressed in [27] by reducing the false positive rate and providing support for more expressive search queries. However, the proposed approach introduces significant overhead with high time complexity. This approach is unsuitable for large-scale or resource-constrained environments. The authors in [28] proposed a dynamic symmetric searchable encryption using an authenticated bloom filter to support efficient updates. The proposed technique is best suited for single-user or tightly controlled environments. Similarly, the authors in [29] proposed a technique based on CP-ABE for fine-grained access, bloom filter for efficient search, and blockchain for fair and trustworthy operations. The proposed technique generates a bloom filter for each file and encrypts both the bloom filter and file using CP-ABE for fine-grained access. The blockchain is used to keep a tamper-proof record of the search history. However, the proposed technique is slow and expensive due to the usage of blockchain for every search, complex because of the three advanced technologies involved, and inflexible for applications that need very fast or frequent searches. The authors in [30] propose a symmetric searchable encryption technique using a combination of B+- tree and Counting Bloom Filter (CBF) for efficient dynamic updates. The proposed technique achieves sub-linear *O*(*logn*) search time. However, this solution has storage overhead due to the B+- tree index and CBFs. The proposed variation requires more bits per element than a standard bloom filter, computational overhead during verification, and significant computational cost. It also introduces additional challenges, such as user revocation, key management, and potential collusion attacks. Finally, the authors in [31] proposed a lightweight dynamic searchable technique by reducing communication overhead on the client side. However, this approach still faces limitations, such as a high false positive rate, a lack of multi-user support, and potential data leakage issues. In a nutshell, the existing bloom filter-based symmetric searchable encryption schemes exhibit one or more limitations, such as communication overhead, elevated time complexity, high false positive rates, and data leakage.

The single keyword non-bloom filter-based search schemes can be categorized as: (a) basic single keyword search [32], (b) order preserving search [33], and (c) fuzzy search [34,35]. In this category, search indexes are generated using one or more methods, such as structure-only index, forward index, bitmap index, inverted index, hash-based index, or cuckoo hashing index. Before uploading encrypted documents to the cloud server, the keywords of each document are extracted and indexed. The encrypted documents and the corresponding indexes are stored on the cloud server. The search query of the data owner/user is transformed into a token before being forwarded to the cloud server. The cloud server uses the received token to search for the encrypted index. Once the server identifies the documents containing the keyword, it returns the matching documents to the data user [36]. Order-preserving search [37] ensures the relative order of the keywords in the encrypted document is maintained while performing the search. However, such schemes are complex, computationally intensive, and have limited security. Fuzzy search schemes enable approximate matches rather than exact matching of search queries [38,39]. This allows the data owner/user to perform a successful search even over a query containing small mistakes, such as incorrect spelling and typographical errors. These schemes are error-resilient and improve the user search experience. However, these schemes are complex, computationally intensive, and encounter a high risk of false positives/negatives [38].

An added advantage of symmetric searchable encryption schemes is its faster search times, making it suitable for large datasets and real-time applications. The use of a single symmetric key in SSE schemes makes query processing more efficient than asymmetric approaches, which have computational overhead due to public-key operations [31]. Therefore, SSE is ideal for scenarios where minimizing latency is essential for handling high query volumes. The summary of the existing symmetric searchable encryption scheme is presented in Table 1.

**Table 1:** Comparison of searchable encryption techniques.

No	Reference	Searching Techniques	Index type	Communication cost	Search time	Key size
1	[10]	Symmetric searchable encryption	Multi-level	0.0938 KBs	0.95 ms	128 bits
2	[4]	Symmetric SE	Structure only	-	1 minute	-
3	[3]	PEKS	Forward index	-	-	-
4	[1]	Symmetric SE	Bitmap	Technique-1: 1,768 KBs Technique-2: 0.42 KBs Technique-3: 0.21 KBs	0.025 ms 0.216 ms 0.115 ms	256 bits
5	[40]	Symmetric SE	Inverted index	-	0.22 ms	128,192,256
6	[41]	Symmetric SE	Inverted index	56 MBs	92.70 s	256 bits
7	[42]	Symmetric SE	Inverted index	-	3.04 ms	256 bits
8	[23]	Symmetric SE	Inverted index	-	601.37 ms	256 bits
9	[43]	DSSE	-	227.82 KBs	3.85 ms	256 bits
10	[44]	SSE	Inverted index	-	-	192 bits
11	[31]	SSE	Forward index	-	0.03 ms	128 bits
12	[45]	SSE	Hash-based	16.72 KB	7.18 s	128 bits

This can be concluded from the above table that the search query time of the LPSI [40,42] is exceptional. In this research study, we are investigating LPSI in detail.

**Fig 2:**
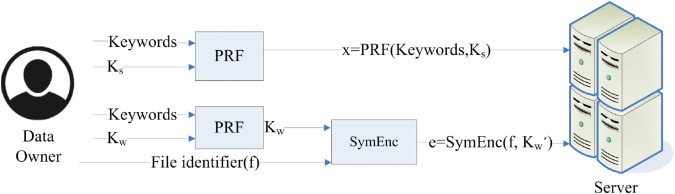
Setup phase.

LPSI enables two different individuals, each possessing a set of components, to simultaneously calculate the intersection without disclosing any further details. The server assigns labels to elements in LPSI, and the data user only obtains these labels when the elements match. OPRF protocols are used to securely generate and match search tokens using symmetric encryption with pseudorandom functions (PRF) [46]. Even in a semi-honest setting, LSE uses LPSI to guarantee privacy by hiding both search and access patterns. LSE can be divided into three steps: setup phase, token generation, and search query. The setup phase refers to the stage where the data owner creates the encrypted database (EDB) that is stored on a cloud server. Fig 2 shows the working of the setup phase.

The data owner generates the secret keys, creates search indexes, encrypts document identifiers, and stores them in the database. The PRF function takes each keyword and converts it to a secure representation. The document identifiers are encrypted using a symmetric encryption scheme. After building the EDB, the data owner uploads it to the cloud server. This process ensures that the data remains confidential while providing the added advantage of searching over encrypted data. It is a critical stage as it lays the groundwork for appropriate secure data access without compromising privacy.

**Fig 3:**
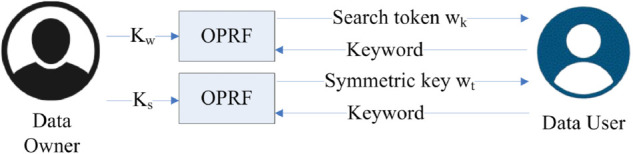
Token generation.

In the token generation phase, the data owner and data user collaborate to prepare a secure search token using an OPRF protocol shown in Fig 3.

This mechanism ensures that the query remains confidential and the data owner gains no knowledge of it [47]. The generated search token enables the data user to execute their query on the cloud server without revealing information about the database contents.

**Fig 4:**
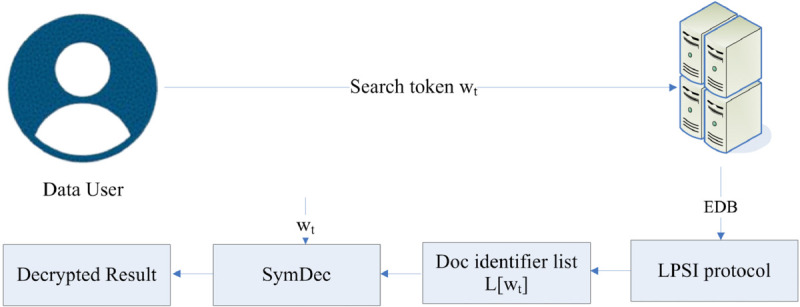
Search query.

Afterward, the data user transmits the search token to the server for performing the search operation. This process is shown in Fig 4.

**Fig 5:**
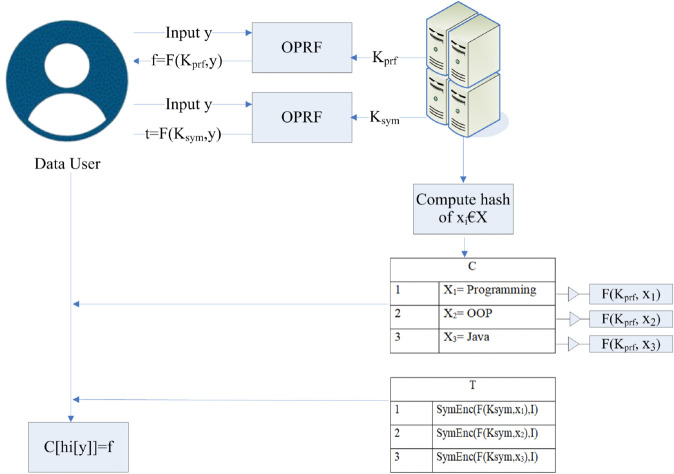
LPSI protocol.

This enables the server to perform the search while remaining oblivious to any information. After transmitting the search token to the server, the server invokes the LPSI protocol for further processing. At the end of this protocol, the data user receives an encrypted list of documents that contain the searched keyword. The data user decrypts the received information using the symmetric key. The detailed process of the LPSI protocol is illustrated in Fig 5. The server selects two keys *K*_*prf*_ and *K*_*sym*_. Afterwards, the server calls the OPRF function twice. In the first round, the data user inputs *y* and the server inputs *K*_*prf*_ and gets *f* = *F*(*K*_*prf*_,*y*). In the second round, the server inputs *K*_*sym*_ and the data user inputs y and gets *t* = *F*(*K*_*sym*_,*y*). This makes it possible for the data user to view the actual records corresponding to the labels.

Afterward, the server maps each element of x∈X into the cuckoo table *C* using the *k* hash function. The *T* table is first initialized by the server, who has a set of labels that match their data. The labels corresponding to each element in *C* are entered into *T*. The server encrypts each label in the T table using a symmetric-key encryption technique. For every label, the sender applies the PRF to the relevant input (such as the element’s identifier) to calculate an encryption key [48]. Then the server sends both the *C* and *T* tables to the data user. With the help of the labels in the *T* table, the data user determines where their set of elements overlaps. During this entire operation, no information regarding the contents of the search query is revealed to the server. The *T* table will be used by the data user to determine which of their items match the labels provided by the server. A collection of labels or identifiers that match the intersection’s elements is sent to the data user [49]. This indicates that the data user is now aware of which of their components correspond to the labels sent by the server. The matched labels associated with the document identifier can then be retrieved by the data user using the C table. Now, the data user has the encrypted identifiers from the C table, data user decrypts them using the decryption key they acquired from the protocols.

According to a recent study, Labeled Symmetric Encryption (LSE) combined with Labeled Private Set Intersection (LPSI) offers an improved solution for searchable encryption, reducing search time on the data user’s side. However, this scheme has a limitation: when the user submits a query to the cloud server, the server generates two tables. These tables contain hash keywords, encrypted file identifiers for the searched keywords, and dummy elements. These tables must then be sent from the cloud to the data user. The size of above mentioned tables is directly linked with the size of the data or keywords. This means that the size of these tables also grows with increases in data or keywords, which increases communication costs. Additionally, the method for generating these tables at the cloud side is inefficient. Cloud service leads to higher costs as resource consumption increases. While the current method performs well with small datasets, it faces significant scaling issues and communication overhead when applied to larger datasets. Another limitation of this technique is that when a data user wants to generate a search token, the data owner needs to always be online. If the data owner is not online at the time of the token generation, the data user cannot perform the search. Therefore, this setup is not feasible for practical applications. Lastly, this approach generates two tables, *S* and *T*, for the entire dataset. In this case, if a data user wants to upload an additional file later, they need to extract the keywords, find the file identifier containing those keywords, calculate the hash of each keyword, and then go to the corresponding locations in S and T to add the information. However, this process is not explained in the research study and appears to be a complex and time-consuming operation. In this research study, we propose a symmetric searchable encryption scheme that addresses the inefficiencies and scalability issues of existing Labeled Private Set Intersection (LPSI), such as high communication overhead, dependency on the data owner being online for token generation, and complex update processes. Moreover, the proposed technique also addresses the common issues of existing bloom filter–based symmetric searchable encryption schemes, such as high false positive rates, high communication and computational overhead, and potential data leakage.

## 3 BloomSec: Scalable and privacy-preserving searchable encryption for cloud environments

**Fig 6:**
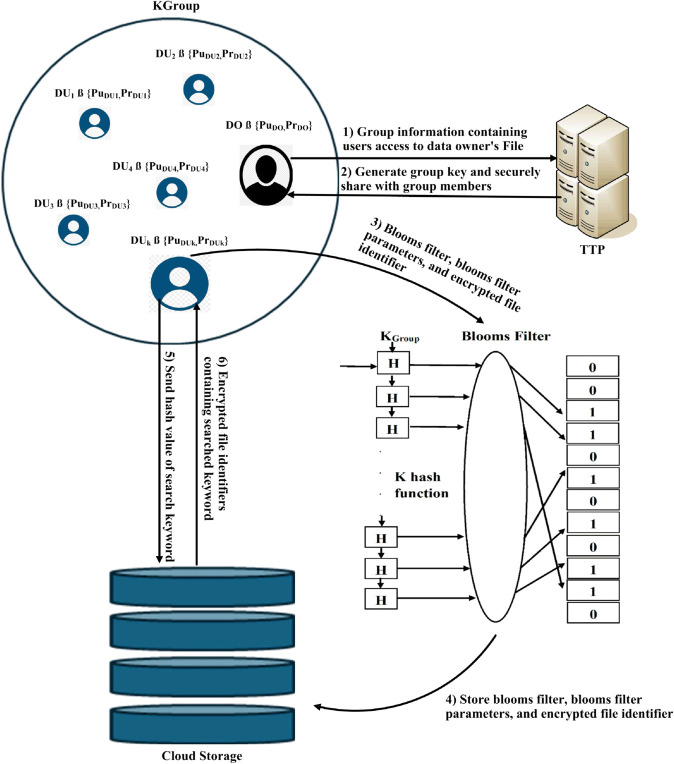
The proposed system model for symmetric searchable encryption.

There are four main entities involved in the proposed symmetric searchable encryption technique: (a) Data User (DU), (b) Data Owner (DO), (c) Trusted Third Party (TTP), and (d) Cloud Storage. In this proposed technique, we assume the Trusted Third Party is trusted, and the cloud is semi-trusted but curious. This means the cloud runs the protocol correctly and may try to learn the secret information. Each Data User and Data Owner possesses a public-private key pair, where only public keys are exchanged between parties, while the private keys remain confidential. DO sends the information of DUs to TTP for the creation of a group that can search on the encrypted data uploaded to the cloud storage by the DO. TTP generates a group key and securely shares it with both the DO and DUs by encrypting it with their respective public keys. Afterward, the DO uses the group key to securely upload data to the cloud storage, and the DUs use that key to search for the encrypted data. The process of the proposed technique is illustrated in Fig 6.

There are three main phases in the proposed symmetric searchable encryption technique: (a) Setup Phase, (b) Token Generation, and (c) Search Query Phase. In the setup phase, DO initially extracts important keywords from each file of the dataset using TF-IDF (Term Frequency-Inverse Document Frequency). Afterward, DO computes the bloom filter parameters based on the total keywords in a file n, which includes the number of hash functions k, the size of a bloom filter m, and a false positive rate p. The process of keyword extraction and bloom filter parameter computation is shown in Algorithm 1 and Algorithm 2.

**Algorithm 1. Extract keywords from file.**

  **Input:** *F*: File

  **Output:** *K*: extracted keywords

1: Content←Read(F) ;

2: TFIDF←Apply_TFIDF(Content) ;

3: K←Sort(TFIDF) ;

4: **return** *K* ;

**Algorithm 2. Find bloom filter parameters.**



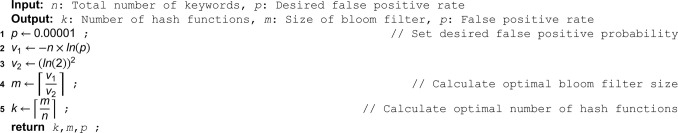



**Fig 7:**
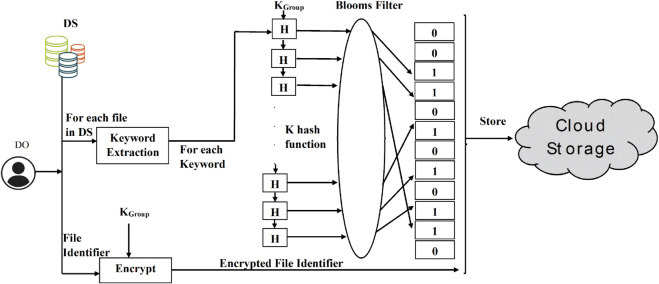
Setup phase.

The diagrammatic representation of the setup phase is shown in Fig 7. For each file in the dataset, a bloom filter(bf) of size n bits is computed by applying k hash functions to each keyword, using Algorithm 3. The first input to the hash function is the concatenation of the keyword and *K*_*Group*_. The subsequent *k*–1 functions are simple hash functions that take the output of the previous hash function as their input. The output of the first key-based hash function is used as input to the second hash function, and this process continues for subsequent functions. Since the size of the hash generated is 256 bits, which is much larger than the bloom filter size, modular reduction by n is performed to ensure that the output falls within the range of 0 to *n*–1. Based on the returned integer hash values, the corresponding positions in the bloom filter (bf) are assigned a value of 1. Afterwards, the corresponding file identifier is encrypted with KGroup and uploaded to the cloud storage along with the corresponding bloom filter (bf), its size (*n*), and the number of hash functions (*k*) for access by DUs. The involvement of KGroup in the generation of the bloom filter ensures that the cloud is unable to guess the keyword in each encrypted file uploaded to the cloud. Algorithm 3 computes the hash of a string and maps it to an integer.

**Algorithm 3. Compute integer hash of string.**



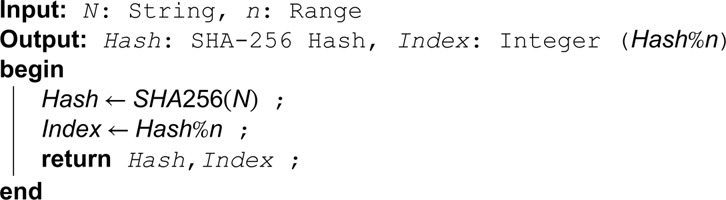



**Algorithm 4. Setup phase for processing dataset.**



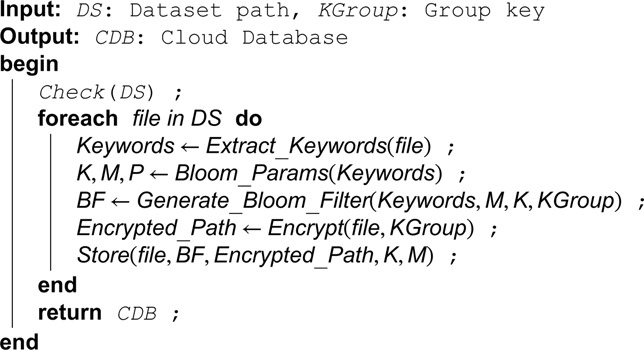



The complete details of the setup phase are shown in Algorithm. 4. The process of setup phase is shown in Fig 7.

Once the data has been uploaded to the cloud storage, DUs can search for the encrypted data. The DU prepares a token, which contains the hash of the search keyword concatenated with *K*_*Group*_, using Algorithm 3, and forwards it to cloud storage.

**Fig 8:**
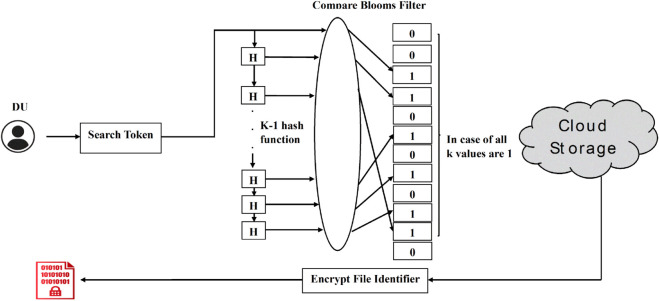
Search query.

In the search query processing phase, for each entry in the cloud database, the values of *k* and *n* are obtained, and *k*–1 hash functions are applied to the received token by performing modular reduction with n. If the bloom filter for a particular file contains 1 at all *k* indexes generated previously, this means the search keyword is available in that file. Hence, the encrypted file identifier is added to the response of this query. This process is illustrated in Algorithm 5. Fig 8 shows the detailed working of search query phase.

**Algorithm 5. Search query processing using token.**



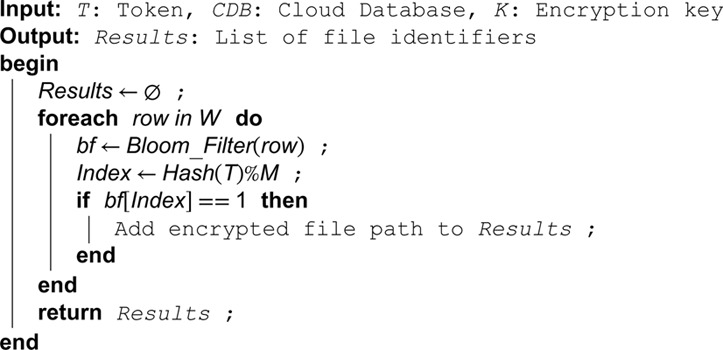



The response of search query phase is detailed in Algorithm 6. The encryption process and decryption process used in the proposed technique is explained in Algorithms 7 and 8.

**Algorithm 6. Decrypt search results.**



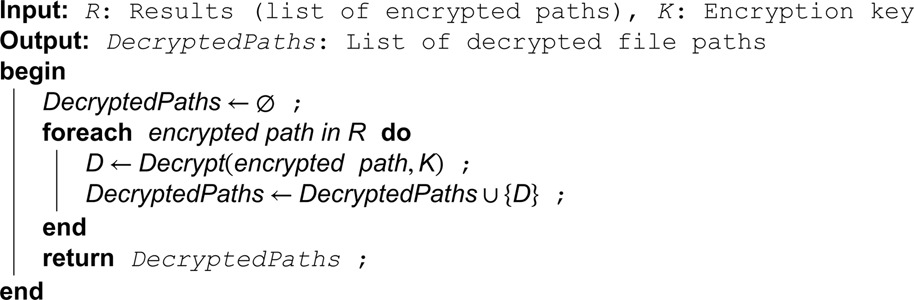



**Algorithm 7. Encrypt data using AES GCM.**



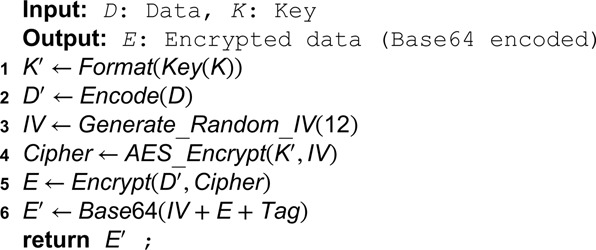



**Algorithm 8. Decrypt data using AES GCM.**



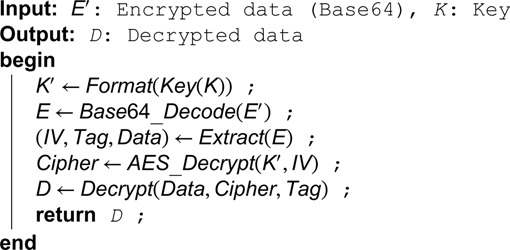



The proposed scheme solves the problem of communication overhead faced in existing schemes by using a bloom filter for each file. A bloom filter can be used to determine if a keyword belongs to a file. It enables fast identity verification while using low memory. When the user submits a search query to the cloud server, the server computes the hashes and compares those hashes with bloom filters. If the values of the bloom filter at the positions of all generated hashes are set to one, it indicates that the searched keyword is present, and the server returns the corresponding encrypted file identifier containing the searched keyword. This way, the communication overhead problem is solved. Moreover, the data user generates the token to perform the search operation, in contrast to the existing schemes where the data owner needs to be always online to generate the token. Lastly, after uploading the entire data set to the cloud, if the data owner wants to upload another file, they only need to extract keywords, compute the hash of keywords, add the keyword to a bloom filter, and upload the bloom filter and corresponding encrypted file identifier to the server.

## 4 Results and discussion

The searchable encryption techniques being studied are implemented using the Python programming language, with performance evaluation carried out on a system with an Intel(R) Core (TM) i5-7300U CPU 2.60GHz, 2.71 GHz. TF-IDF (Term Frequency-Inverse Document Frequency) is used to extract keywords from files, AES-256 is used for encryption, and SHA-2 is used for hashing. The subset of Enron email dataset [50,51] of size 250MB, consisting of 109789 files, is used in the evaluation. The evaluation parameters are operational time, storage cost, and communication cost. To ensure baseline fairness, all experiments were conducted using identical hardware and software configurations. The details are given in Table 2.

**Table 2:** Hardware and software configurations.

Category	Component	Version/Details
Operating System	Windows 11	Version 24H2
Development Language	Python	3.10.12
Library	hashlib	3.10
	Os	3.10
	Crypto (PyCryptodome)	3.20.0
	scikit-learn	1.3.0
	Openpyxl	3.1.2
	Numpy	1.25.2
	base64, math	Built-in
Hardware	Core (TM) i5-7300U CPU	2.60GHz, 2.71 GHz. TF-IDF
Threading / Parallelism	All operations, such as file traversal, keyword extraction, encryption are single-threaded.	
System Load	The setup is not resource-intensive (mostly file I/O and memory-based processing).	
Communication	Communication is simulated.	
Encryption Details	Uses AES-GCM (AES in Galois/Counter Mode) from PyCryptodome	
Encoding	base64	

### 4.1 Operational time

Total operational time of LISP protocol [40] is the sum of the time to extract keywords from all files (*T*_*ek*_), the time to generate a hash of keywords (*T*_*x*_), the time to encrypt the corresponding file identifier (*T*_*e*_), and the time to upload data on the cloud (*T*_*c*_).

$$\text{Setup Time}=T_{e}k+T_{x}+T_{e}+T_{C}$$ (1)

The total operational time required to complete the token generation phase can be computed by adding the time to generate the value wt, the time to generate the value of *w*_*k*_, and the time to communicate the value of *w*_*k*_ and *w*_*t*_. This can be represented using the following formula:

$$\text{Token Generation Time}=T_{wt}+T_{wk}+T_{C}$$ (2)

The total operational time required to complete the search query phase can be computed by adding the time to find the value of *t* and *f*, the time to calculate the hash of *x*, the time to calculate the *C* table, the time to calculate *T* table, and the time to communicate the value of *tf*, *hash*_*x*_, *C*, and *T*. This can be represented using the following formula:

$$\text{Search query Time}=T_{tf}+T_{\left(hash\_x\right)}+T(C\_table)+T(T\_table)+T_{C}$$ (3)

Similarly, the total operational time required for the proposed scheme is the sum of the time to extract keywords from all files (*T*_*ek*_), time to calculate the bloom filter (*T*_*m*_), time to encrypt the corresponding file identifier (*T*_*e*_), and time to upload data on cloud (*T*_*C*_). The total operational time required to complete the setup phase can be computed using the following formula

$$\text{Setup time}=T_{ek}+T_{m}+T_{e}+T_{C}$$ (4)

In the proposed technique, the search token is directly generated by the data user by performing a hash on the search query once and forwarding it to the cloud server. The total operational time required to complete the search query phase can be computed by adding the time to compute the hash *k*–1 times (*T*_*HK*_) and the time to match the *k* hash values with the stored bloom filter of each file (*T*_*Match*_). This can be represented using the following formula:

$$\text{Setup time}=T_{ek}+T_{m}+T_{e}+T_{C}$$ (5)

### 4.2 Communication cost

The communication cost of the LPSI protocol [40] during the setup phase depends on the values of *x* and *e*. The number of bytes sent to the cloud is determined by the sizes of *x* and *e*. The total bytes to be transmitted is the sum of the size of *x* and the size of *e*. This can be represented using the following formula:

$$\text{Setup Communication cost}=(Size_{x}+Size_{e})\times n$$ (6)

In the token generation phase, the search token *wt* and symmetric key *wk* are sent to the data user, so the communication overhead depends on the sum of the size of *wt* and *wk*. This can be represented using the following formula:

$$\text{Token generation Communication cost}=Size_{wt}+Size_{wk}$$ (7)

In the search query phase, the *C* and *T* tables are sent to the data user, so the communication overhead depends on the sum of the sizes of *C* and *T*. This can be represented using the following formula:

$$\text{Search query Communication cost}=Size_{C}+Size_{T}$$ (8)

The size of *C* and *T* are computed using the following formulas:

$$Size_{C}=n\times 256bits$$ (9)

$$Size_{T}=fid_{AVG}\times n\times S_{efid}$$ (10)

Similarly, the communication cost for the proposed scheme is calculated. During the setup phase, the values of the bloom filter parameters and the bloom filter will be uploaded to the cloud. The number of bytes sent to the cloud will depend on the sizes of the bloom filter parameters and the BF. The total bytes to be sent is the sum of the size of the bloom filter parameters and the size of the BF. This can be represented using the following formula:

$$\text{Setup Communication cost}=Size_{K}+Size_{BF}$$ (11)

In the proposed technique, the search token is directly generated by the data user by performing a hash on the search query once and forwarding it to the cloud server. Hence, the communication cost of the token generation phase is zero, as this phase does not involve communication but only computes the hash value for the search query. In the search query phase, the search is performed on the bloom filter, and the file identifiers of the files containing the search keyword are returned. Therefore, the communication overhead depends on the total number of file identifiers that contain the search keyword. This can be represented using the following formula:

$$\text{Search query Communication cost}=\sum_{i=1}^{V}Sizeof(fid_{i})$$ (12)

### 4.3 Storage cost

The total number of keywords extracted from the dataset files is represented by *n*. In LPSI, multiple file identifiers are associated with a keyword. Let the total number of identifiers stored on the cloud server be represented by *t*. We can compute the average number of file identifiers stored with each keyword using the following formula:

$$fid_{AVG}=\frac{t}{n}$$ (13)

In the setup phase of LPSI [40], the hash value of the keyword, represented by *x*, and the corresponding encrypted file identifier, represented by *e*, are stored on the cloud server. The HMAC based on SHA-2 is used to generate a 256-bit hash value, and AES with a 128–*bit* key is used for encryption. The total storage cost required to store the dataset can be computed using the following formula:

$$\text{Storage cost}=n\times 256\text{ bits}+fid_{AVG}\times n\times S_{efid},$$ (14)

Where *S*_*efid*_ represents the size of a single encrypted file identifier. In the token generation and search query phase, there will be no data stored on the cloud.

To calculate the storage cost required for the proposed scheme, we need to consider the following components the bloom filter size, bloom filter parameters, and Encrypted file identifiers. The size of a bloom filter depends on the number of words (*n*) and the false positive rate (*p*). Each file identifier is stored in encrypted form. The size of each encrypted file identifier depends on the encryption scheme used (e.g., AES-128). The size of a bloom filter (in bits) for each file is calculated using the formula:

$$m=\frac{-n\times ln(n)}{(ln(2){)}^{2}}$$ (15)

Where *n* is the number of words in the file and *p* is the false positive rate. Now convert the size of the bloom filter into bytes.

$$\text{Size of }(bf_{i})=\frac{m}{8}$$ (16)

$$TS_{efids}=N\times S_{efid}$$ (17)

Where *TS*_*efids*_ represents the size of all encrypted file identifiers and *S*_*efid*_ represents the size of a single encrypted file identifier.

If there are *N* files in the dataset, and each file has its own bloom filter, the total storage for bloom filters is:

$$\text{Size of Bloom Filters}=\sum_{i=1}^{N}Sizeof(bf_{i})$$ (18)

The total storage cost required for the proposed scheme is the sum of the total size of a bloom filter and the total size of an encrypted file identifier.

$$\text{Storage cost}=\sum_{i=1}^{N}Sizeof(bf_{i})+N\times TS_{efids}$$ (19)

**Fig 9:**
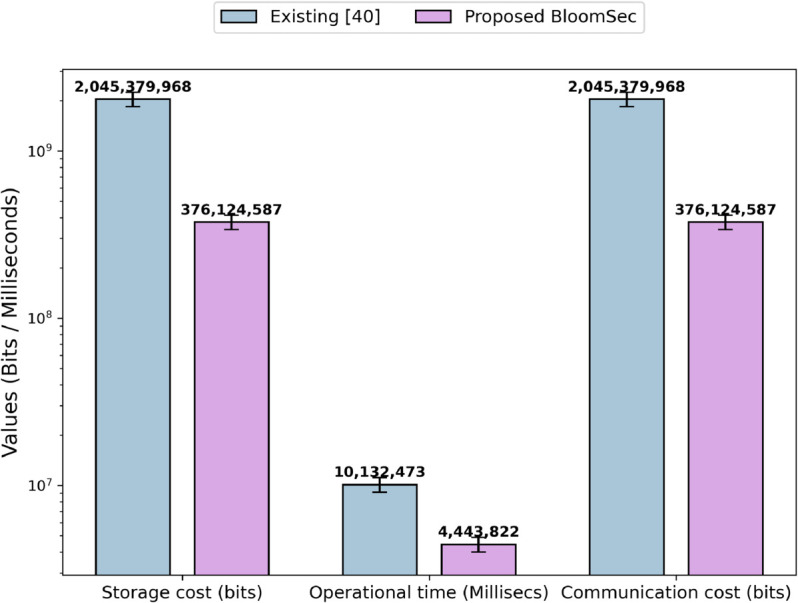
Storage cost, operational time, and communication cost of the setup phase.

In the token generation and search query phase, there will be no data stored on the cloud. Fig 9 shows the storage cost in bits, operational time in milliseconds, and communication cost in bits. The results demonstrate that the performance of the proposed technique, with respect to communication cost, operational time, and storage cost, is better compared to the existing technique [40]. The improved performance of the proposed technique is due to the fact that it requires fewer resources compared to the existing technique. In the existing technique [40], the evaluation parameters are directly associated with the keywords and files in the dataset. As the number of keywords or files in the dataset increases in both cases, the communication cost, storage cost, and operational time also increase. This is because the existing technique stores file identifier information for each keyword. In our experimental setup, an average of 34 file identifiers is stored for each keyword. If we increase the number of files, the number of file identifiers for each keyword will increase, which ultimately raises the communication cost, storage cost, and operational time. Similarly, if the number of keywords in a file increases, the existing scheme stores all file identifiers containing those keywords, which ultimately increases the evaluation parameters discussed above. In contrast, the proposed technique stores a bloom filter along with its parameters and the corresponding encrypted identifier for each file in the dataset. Firstly, the size of the bloom filter is only dependent on the number of keywords in a file; it does not depend on the number of files in the dataset. Secondly, the increase in evaluation parameters, due to the rise in keywords, is considerably lower compared to the existing technique.

**Fig 10:**
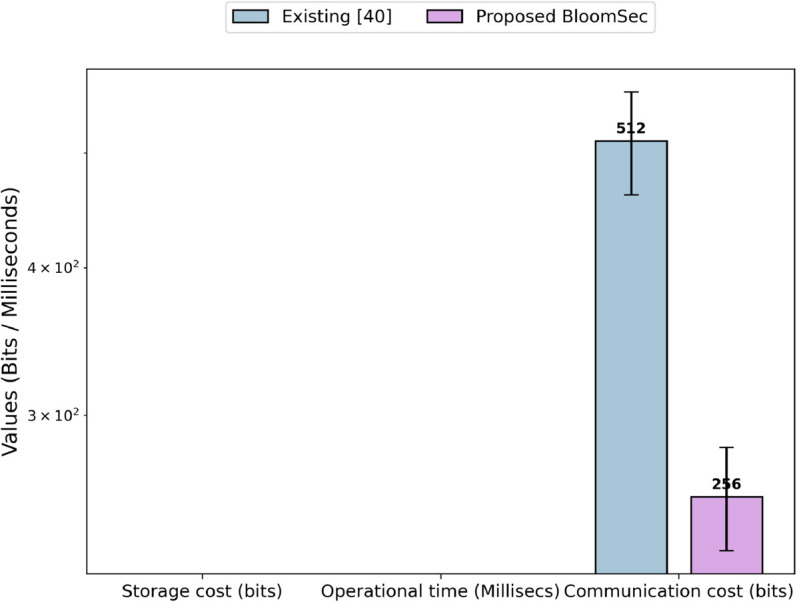
Storage cost, operational time, and communication cost of token generation.

Fig 10 shows the values of the evaluation parameters in the token generation phase. As both techniques generate a few values temporarily and do not store any information on the server, the storage cost for both in the token generation phase is zero. During the token generation phase, the existing technique [40] computes the search token, represented as wk, and the symmetric key, represented as wt, while the proposed technique generates the search token by applying a key-based hash function to the search keyword. The operational time required to compute these values in both techniques is less than one millisecond. Since the measurements are in milliseconds, the result is displayed as zero. Similarly, the communication cost of the token generation phase for the existing technique [40] is 512 bits, as both wk and wt have a size of 256 bits each, while the proposed technique has a communication cost of 256 bits due to the search token generated, as discussed above.

**Fig 11:**
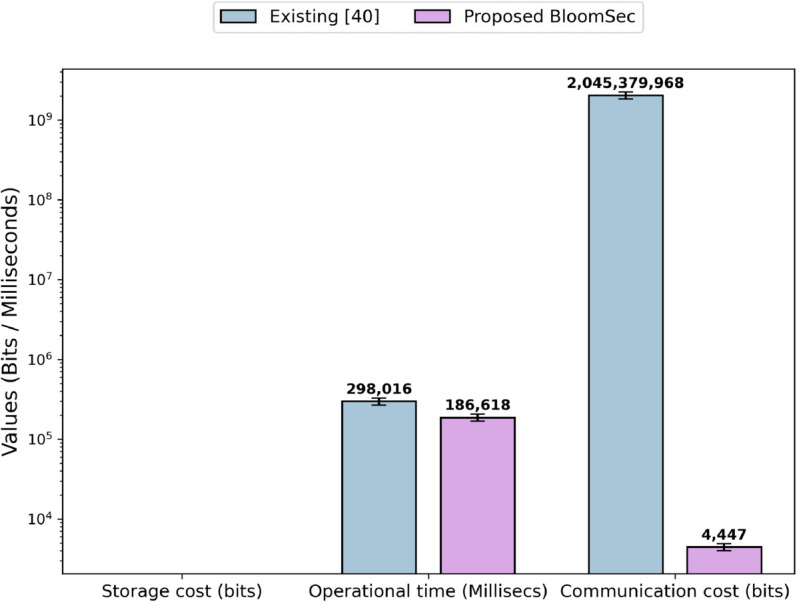
Storage cost, operational time, and communication cost of a search query phase.

Fig 11 shows the results of the search query phase. The storage cost of the search query phase is zero because all the information generated during this phase is temporary and is not stored on the server. The performance of the proposed technique is better than the existing technique [40] in operational time and communication cost. The existing technique [40] generates two tables C and T during the search query phase. The total entries in the C and T tables are equal to the number of keywords. Each entry in the C table is 256 bits in size, representing the hashed value of a keyword, while the T table contains the corresponding encrypted file identifiers that include that keyword. As discussed above, an average of approximately 34 file identifiers are stored for each keyword. This information needs to be forwarded to the data users. As the volume of data or keywords increases, the size of these tables also grows, thereby increasing communication costs. In contrast, our proposed technique only forwards the encrypted file identifiers containing the search keyword, thereby significantly reducing the communication cost. Similarly, the operations involved in completing the search query phase of the existing technique require more time compared to the proposed technique.

The experiments are repeated 15 times to compute the confidence intervals. The error bars indicate the 95% confidence intervals for each metric, representing the statistical reliability of the measurements. The smaller margin of error shows higher precision and results consistency.

### 4.4 Formal SSE leakage model and security proof

Suppose dataset 𝒟 contains *N* files, where the *i*^*th*^ file is denoted by ${ℱ}_{i}$. Multiple keywords are extracted from each ${ℱ}_{i}$ and represented as ${𝒲}_{i}=\{{\hat{w}}_{1},{\hat{w}}_{2},{\hat{w}}_{3},{\hat{w}}_{4},\ldots \}$. The data owner generates a bloom filter $\beta_{i}$ based on the corresponding keyword set ${𝒲}_{i}$ for each file ${ℱ}_{i}$. The parameters (*m*,*k*) used by bloom filters represent the size of the bloom filter *m* and the number of independent hash functions *k*, respectively. The data owner encrypts each file identifier $\text{ID}({ℱ}_{i})$ using symmetric encryption ${\text{Enc}}_{gk}$. Thus, the cloud stores the tuple for each file $\left\{\beta_{i},{\text{Enc}}_{gk}(\text{ID}({ℱ}_{i})),m,k\right\}$. An authorized data user searches for a keyword $\hat{w}$ by generating a token ${𝒯}_{w}=\{h_{1}=H(\hat{w}||gk),h_{2}=H(h_{1}),h_{3}=H(h_{2}),\ldots ,h_{k}=H(h_{k-1})\}$, where *H* denotes a standard one-way hash function. The cloud verifies each $\beta_{i}$ by checking if all bits indexed by ${𝒯}_{w}$ are set. If true, the encrypted file identifier ${\text{Enc}}_{gk}(\text{ID}({ℱ}_{i}))$ is returned. The leakage function Λ is defined as:

The leakage function for the setup phase ($\Lambda_{\text{setup}}$) reveals the bloom filter ($\beta_{i}$), encrypted file identifier (${\text{Enc}}_{gk}(\text{ID}({ℱ}_{i}))$), and bloom filter parameters *m* and *k*. Similarly, the leakage function of the search query phase ($\Lambda_{\text{query}}$) reveals whether two queries are associated with the same token ${𝒯}_{w}$ and access pattern which encrypted file identifiers are returned for each query. No additional information about the search query and file identifiers is exposed.

Assume one-way hash function (*H*) and symmetric encryption (${\text{Enc}}_{gk}$) algorithms are secure, any probabilistic polynomial-time adversary (𝒜) is not able to learn any information about the file identifiers ($\text{ID}({ℱ}_{i})$) and search keyword ($\hat{w}$) beyond the leakage Λ. For polynomial-time adversary (𝒜), there exists a simulator that, given only Λ, can simulate 𝒜’s view such that:


|Pr[𝒜 outputs 1∣RealView]−Pr[𝒜 outputs 1∣SimulatedView]|≤negl(λ)

where *λ* is the security parameter and negl(·) is negligible.

The file identifiers are encrypted using a secure encryption technique, so they do not reveal any information about the file identifiers themselves. Additionally, the bloom filter is generated based on the group key, which prevents it from leaking any information about the keywords of the files. Therefore, both components make the setup phase indistinguishable. Search query indistinguishability is achieved by applying an irreversible one-way hash function to the search query concatenated with the group key (GK), thereby hiding the plaintext keyword during the search query phase.

### False positive analysis and impact

To compute the false positive, we have used the following experimental setting: the false positive rate *p* = 10^−5^, total search queries =1,000,000, average one-way hash function *k* applied 17, and the average number of files indexed by each query *N*_*f*_ = 128. The false positive rate can be computed using the following formula:

$$\text{FP per query}=p\times N_{f}=10^{-5}\times 128=0.00128$$ (20)

This shows that there is only one false positive out of 781 queries; hence, the total number of false positives over 1,000,000 queries can be computed as:

Total FP=0.00128×1,000,000=1,280 (21)

The expected number of false positives is 1,280 over 1 million queries with this setting. Precision can be computed using the following formula, where *t* is the average number of true positive results per query:

$$\text{Precision}=\frac{t}{t+\text{FP per query}}=\frac{127}{127+0.00128}\approx 0.9999899\approx 99.99\%$$ (22)

Recall remains 100% since bloom filters don’t cause false negatives.

## 5 Conclusion

A few essential characteristics necessary for a good searchable encryption scheme are robust security measures to prevent unauthorized access and effective search capabilities to manage big datasets. The proposed scheme uses a bloom filter, which is space-efficient, and provides robust security. This means that if we apply the hash function on the keyword multiple times and match it to the filter, it becomes difficult for an attacker to determine the original keyword even if the attacker has access to the bloom filter. To determine whether the keyword is present in the bloom filter, it offers constant time operation complexity. It allows encrypted keyword searches to be performed quickly, regardless of large datasets. The results demonstrate that, in comparison with existing symmetric searchable encryption techniques, the proposed method using bloom filters provides significantly improved results. bloom filters are not error-free, even though they enhance privacy and reduce data disclosure. False positive results from bloom filters could result in irrelevant information recovery. False positive results from bloom filters can lead to the recovery of irrelevant information. The value of p determines the false positive rate, and in our proposed technique, we set the value of p to 0.00001, which does not significantly affect the output. Alternatively, while hiding accurate matches, it may be used for a privacy benefit. Sometimes efficiency requires controlled leaking, such as letting the cloud server learn what files matched a query. Finding an acceptable balance between privacy standards as well as such vulnerability is difficult.

In the future, we will revise the proposed scheme and introduce the concepts of forward secrecy and backward security whenever there is a change in group members. Moreover, we will extend the proposed technique to support searching on multiple keywords and fuzzy search features.
